# Effects of Plyometric and Balance Training on Neuromuscular Control of Recreational Athletes with Functional Ankle Instability: A Randomized Controlled Laboratory Study

**DOI:** 10.3390/ijerph18105269

**Published:** 2021-05-15

**Authors:** Pi-Yin Huang, Amornthep Jankaew, Cheng-Feng Lin

**Affiliations:** 1 Department of Physical Therapy, College of Medicine, National Cheng Kung University, Tainan 70101, Taiwan; piyinhuang@gmail.com; 2 Institute of Allied Health Sciences, College of Medicine, National Cheng Kung University, Tainan 70101, Taiwan; amornthep.j330@gmail.com; 3 Physical Therapy Center, National Cheng Kung University Hospital, College of Medicine, National Cheng Kung University, Tainan 70101, Taiwan

**Keywords:** proprioception, ankle sprain, landing, muscle activity

## Abstract

Plyometric exercise has been suggested for knee injury prevention in sports participation, but studies on ankle plyometric training are limited. This study aims to investigate the change of joint position sense and neuromuscular activity of the unstable ankle after six-week integrated balance/plyometric training and six-week plyometric training. Thirty recreational athletes with functional ankle instability were allocated into three groups: plyometric group (P) vs. plyometric integrated with balance training group (BP) vs. control group (C). Ankle joint position sense, integrated electromyography (EMG), and balance adjusting time during medial single-leg drop-landing tasks were measured before and after the training period. Following the six-week period, both training groups exhibited a lower absolute error in plantar flexion (P group: pre: 3.79° ± 1.98°, post: 2.20° ± 1.31°, *p* = 0.016; BP group: pre: 4.10° ± 1.87°, post: 2.94° ± 1.01°, *p* = 0.045), and the integrated group showed a lower absolute error in inversion angles (pre 2.24° ± 1.44° and post 1.48° ± 0.93°, *p* = 0.022), and an increased integrated EMG of ankle plantar flexors before landing. The plyometric group exhibited a higher integrated EMG of the tibialis anterior before and after landing (pre: 102.88 ± 20.93, post: 119.29 ± 38.33, *p* = 0.009 in post-landing) and a shorter adjusting time of the plantar flexor following landing as compared to the pre-training condition (pre: 2.85 ± 1.15 s, post: 1.87 ± 0.97 s, *p* = 0.006). In conclusion, both programs improved ankle joint position sense and muscle activation of the ankle plantar flexors during single-leg drop landing. The plyometric group showed a reduced adjusting time of the ankle plantar flexor following the impact from drop landing.

## 1. Introduction

Ankle sprain injuries are the most common lower limb injuries among physically active individuals and athletes [1]. The injured ankle joint can cause joint receptors damage and chronically alter motor control. Individuals with a history of ankle sprains are reported with sensorimotor impairments corresponding to functional ankle instability (FAI), defined as subjective ankle instability, giving way, and repetitive ankle sprain [2]. Patients with FAI have highlighted motor control changes observed via electromyography (EMG) during walking and drop-landing tasks [3]. The simultaneous onset of muscle activation of the peroneal muscle, tibialis anterior, and lateral gastrocnemius was found in athletes with FAI during a landing task; however, the peroneal muscle activates first followed by the lateral gastrocnemius, and the onset of tibialis anterior has close to the ground contact in the healthy individuals [4]. Furthermore, individuals with FAI had a longer time to stabilization as compared to healthy people when performing a jump with single-leg standing [5]. All of these reveal that balance control deficit and lack of designated muscle activation sequence during landing movement exist in an individual with ankle instability leading to degraded functional performance [6] and movement confidence due to the fear of re-injury [7].

Balance training is an effective way of reducing episodes of inversion in individuals with chronic or functional ankle instability and improving joint position error [8]. Both three-week [8] or six-week [9] balance training had been demonstrated not only to reduce ankle joint position error but also improve postural stability. However, the effects of balance training on the muscle activation strategy of individuals with or without FAI are less clear. A study found that healthy participants did not have significant change in the onset of peroneal muscle activity in a sudden inverted ankle movement after a four-week balance training program [10]. Similarly, for individuals with FAI and previous multi-station proprioceptive training, the integrated EMG signals of the peroneus longus and brevis and tibialis anterior showed no change for the first 60 ms following a sudden external ankle inversion stimulus [11].

Plyometric training involves a series of stretch-shortening cycle (SSC) movements designed to induce the repeated lengthening and shortening of various muscle-tendon complexes, and it also includes various types of body-weight jumping exercises, including drop-jumps, countermovement jumps, squat jumps, leg bouncing, and hopping [12]. Plyometric exercise is recognized as an effective strategy for “reactive neuromuscular training” since it changes the motor unit recruitment pattern and muscle activity by facilitating the sensorimotor system [13] and increases the excitability of the neurological receptors, thereby improving the re-activity of the neuromuscular system. Furthermore, plyometric exercises targeted at the shoulder joint [14] or the knee joint [15] enhance corresponding joint proprioception by facilitating neural adaptations. After six-week plyometric training, Chimera et al. (2004) reported that it not only increased muscle activation but also increased muscle co-activation in the preparatory phase during drop jumping [16]. Previous studies have shown that plyometric exercise reduces the risk of injury by increasing the functional stability of the lower limb joints [15]. Thus, Alikhani et al. (2019) suggested that plyometrics should be incorporated into the training regimens of athletes in order to reduce the incidence of serious knee injuries [15].

Athletes with ankle instability mainly demonstrate alteration of joint kinematics and neuromuscular control and proprioception loss, leading to recurrence of ankle sprain [17]. Due to the pathomechanics of the associated ligaments injury, the mechanical instability and functional instability can influence sports activities especially in running, jumping, and cutting motions sports leading to diminished athletic performance [18]. Successful impairment-based rehabilitation programs aimed at participants returning to normal sports activities without adversely affecting sports performance and skill need to be emphasized [19]. Even though the updated evidence obviously shows that plyometrics improve neuromuscular control in uninjured individuals through the facilitation of the neurological receptors, such as the muscle spindle, the Golgi tendon organ, and receptors around the joint capsule and ligament [20], the feasibility and effects of plyometric training for the neuromuscular rehabilitation of athletes with FAI are unclear. Additionally, combination of plyometric and balance training in healthy adolescents (age 12–15 years old) showed superior results than plyometric training alone in leg stiffness and running performance. Their findings highlighted that the combined training can mitigate the impacts of isolated plyometric training through the reduction of the stretch-shortening stress on the neuromuscular system [21]. However, the effects of isolated plyometric training and that combined with balance training on neuromuscular adaptation have not been directly compared among athletes with FAI. Thus, the purpose of this study is to investigate the effects of a six-week plyometric training program and a six-week integrated balance/plyometric training program on the proprioception performance and neuromuscular control of recreational athletes with ankle instability. In performing the study, it is hypothesized that both training regimens improve the joint position error, minimize the time required to stabilization, and increase the EMG activity of ankle muscles.

## 2. Materials and Methods

### 2.1. Study Design

A randomized controlled laboratory trial was used in this study. The data collection was conducted at a university motion analysis laboratory. Athletes were recruited from flyers, online advertisement, and direct contact with sport teams in the university. Participants were blinded to the study group and randomly assigned via sealed opaque envelopes. All of the recruited participants were informed of the purpose of the present study and the associated experimental procedures and were requested to read and sign a consent form prior to participation. The study procedures and consent forms were approved by the Institutional Review Board of University Hospital (Ethical approval code: ER-98-103) and the study was listed on the ISRCTN registry with the study ID ISRCTN16780192 (Supplementary Table S1).

### 2.2. Participants

A G*Power 3.1.9.7 program (Heinrich-Heine-Universität Düsseldorf, Düsseldorf, Germany), a statistical power analysis program for biomedical science, was used to compute the sample size required in this study. The sample size was calculated based *n* pilot data. The calculated effect size is 0.8 (large effect size). To achieve 80% statistical power with an alpha level of 0.05 for a dependent mean study design, at least 26 participants were required.

**Figure 1:**
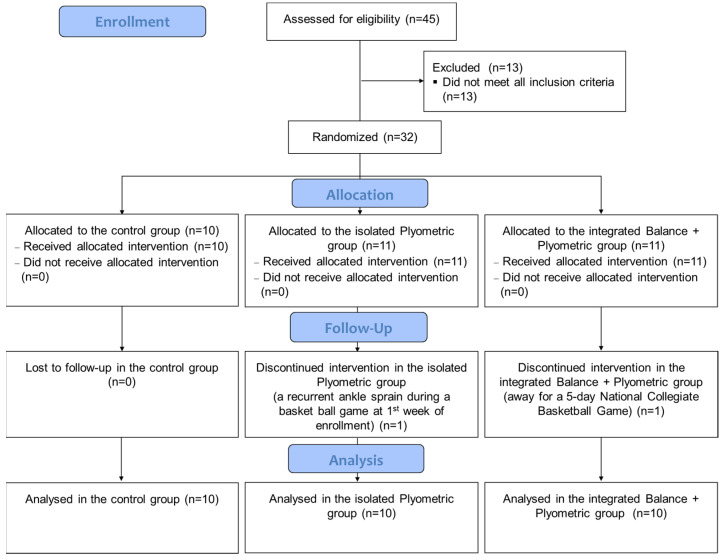
The flow diagram of recruited participants.

Forty-five collegiate recreational athletes aged between 18 and 30 years old, all with FAI [22], were recruited from local campuses at initial recruitment. After initial physical examination, 13 athletes were excluded from this study. The remaining athletes were randomly divided into three groups, namely isolated plyometric (P, 9 males and 2 females), integrated balance + plyometric (BP, 9 males and 2 females) and control (C, 7 males and 3 females). One male athlete in the plyometric group and one male athlete in the integrated group dropped out during training (Figure 1). All of the participants were screened using the Cumberland Ankle Instability Tool (CAIT) questionnaire [23], and a score lower than 24 indicated severe instability [24] (Table 1). The inclusion criteria were specified as follows: (a) participate regularly (at least 1–2 h each time, 2–3 times per week) in sports activities (e.g., basketball, rugby, soccer, and volleyball); (b) have experienced at least one prior ankle inversion sprain that results in swelling, pain, and dysfunction over the past 12 months; (c) have experienced multiple ankle sprains or ankle “giving way” events over the past 12 months; (d) score less than 24 on the CAIT questionnaire [24]; and (e) clinically test negative in anterior drawer and talar tilt tests. The clinical tests were performed by two licensed physical therapists with at least 3 years clinical experience. For those participants with history of bilateral ankle sprains, the ankle with the lower CAIT score was chosen for testing. Applicants with any form of neurological disorder or lower extremity injuries that would affect balance or an ankle sprain within a month were excluded.

### 2.3. Experimental Procedure

**Figure 2:**
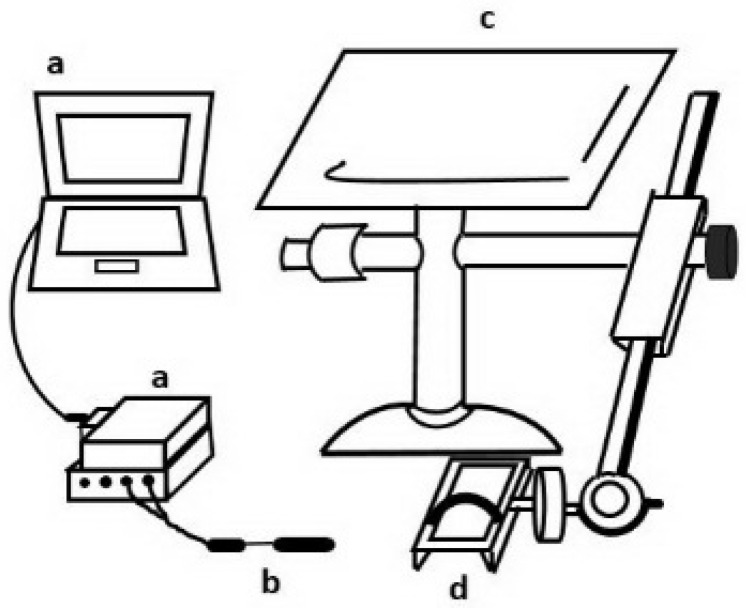
Schematic illustration of joint position test. a: data acquisition system; b: a two-axis electrogoniometer; c: custom-made chair and instrument; d: footplate.

Joint position sense test. The ankle joint proprioception of the participants was evaluated by means of an electrogoniometer (SG 150, Biometrics, UK). The reliability of the ankle joint proprioception test among recreational athletes with ankle instability was reported as ICC = 0.94–0.98 [25]. During the test, the participants were asked to sit in an upright position on a custom-made chair such that both hip and knee joints were flexed at 90° (Figure 2). The ankle was then strapped to a footplate in a neutral position such that the axis of the ankle motion in the sagittal plane was aligned with a line connecting the lateral and medial malleoli. The axis of the ankle motion in the frontal plane was then re-aligned with a line connecting the heel and big toe in order to measure inversion-eversion position sense. The participants were asked to close their eyes in order to avoid any visual effects and were then requested to move their ankle to one of four different ankle joint positions, namely 10° dorsiflexion, 10° eversion, 15° plantar flexion, and 15° inversion. The participants received a standard instruction, “try to move your ankle back to the same position as I just moved to” and were given 3 practice trials in random directions to ensure they understood the procedure. A 5-min resting period was given between practice trials and collected trials. All of the tests started from ankle neutral position. The resulting ankle joint angle was measured using a twin-axis electrogoniometer (SG 150, Biometrics, UK), which can measure motions of two planes at the same time. The electrogoniometer was attached to the lateral ankle joint with one axis parallel to the fibula and one axis parallel to the 5th metatarsal bone to measure the plantar-dorsiflexion angle. Each position was tested three times in a random sequence.

Single-leg medial drop-landing test. A single-leg medial drop-landing task with excellent reliability (ICC = 0.97) was chosen because that task causes greatest inversion/eversion angles, which are more challenging for athletes with FAI [25]. For each trial, the participants were asked to maintain a single-leg stance with both hands on the waist for 3 s as a preparatory movement. In response to an auditory cue, the participants then hopped down onto a force plate; regained their stability as quickly as possible and kept their body erect and the head looking forward. Once the participants reached a balance status, the participants were requested to maintain the single-leg stance for another 5 s. A failure was defined as repetitive hopping on the force plate, the unloaded foot contacting the ground, or moving the hands away from the waist.

The muscle activity signal throughout the drop-landing test was measured using a surface EMG system (Myomonitor, Delsys Inc., Boston, MA, USA) at a sampling rate of 2000 Hz and common mode rejection ratio of 90 dB. In accordance with the recommendations of a previous study [26], the bipolar electrodes were placed on the muscle bellies of the tibialis anterior (TA), peroneal longus (PL), gastrocnemius medialis (GM), gastrocnemius lateralis (GL), and soleus (SOL) of the unstable leg. To minimize skin impedance, the skin was shaved and cleaned with alcohol before the electrodes were attached. A voluntary contraction test was performed to confirm the correct placement of each electrode. Note that to ensure experimental consistency, the same investigator placed the electrodes for all the participants.

### 2.4. Data Reduction

Joint position sense. The joint position sense before and after training was evaluated with absolute error, defined as the absolute difference (in degrees) between the measured angle (i.e., the angle perceived by the participant) and the target position. The average absolute error over three trials was presented for each angle tested.

Single-leg drop landing. The drop-landing task was partitioned into two phases for purpose of measurement and analysis, namely a pre-landing phase and a post-landing phase. The pre-landing phase corresponds to the 200-ms period immediately prior to contact with the force plate while the post-landing phase corresponds to the 200-ms period immediately after contact with the force plate [4]. In both phases, the raw EMG signals were processed using a 4th-order Butterworth band-pass filter at 40~400 Hz and then full-wave rectified to generate linear envelope. The muscle activity was then quantified by calculating the area under the linear envelope voltage curve, so-called integrated EMG, and expressed as the percentage of peak activity related to the linear envelope (% • ms) [27].

**Figure 3:**
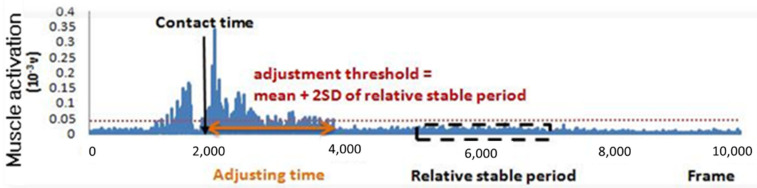
Calculation of adjusting time parameter in landing test (an example of ankle dorsiflexors).

The time required for each individual to regain single-leg balance following landing was evaluated using a novel adjusting time parameter, defined as the time from the moment of foot contact to the moment at which the muscle activity level fell to a value lower than the mean + 2 SD value of the relatively stable period (Figure 3). The adjusting time parameter was separately determined for three muscle groups, namely the ankle dorsiflexors and the ankle plantar flexors. The dorsiflexor EMG was computed as the rectified EMG of the TA muscle. Meanwhile, the plantar flexor EMG was evaluated as the summated rectified EMG of the GM, GL, and SOL muscles.

### 2.5. Training Programs

The 30 participants were randomly assigned to the plyometric group (P), integrated balance training/plyometric training group (BP), and control group (C). Each training program lasted for 6 weeks with 3 individual sessions per week [28]. Each individual was requested to participate in at least two-thirds of the sessions (i.e., 12 of the 18 sessions) and was advised that failure to do so would result in their exclusion from the study. A licensed physical therapist trained and supervised all the participants in the training period and properly adjusted the intensity (i.e., repetitions and movement speed) of the training protocol depending on the ability and performance of each participant. The content of plyometric training started with a simple squat jump and progressed to a challenging jump and hops, while the integrated balance training/plyometric training group involved jumps and a balanced squat or balanced lunge every week. The complete training program can be found in Huang et al. [28] and Supplementary Table S2.

### 2.6. Statistical Analysis

The group demographics (age, body height, body mass) were compared by means of a one-way analysis of variance (ANOVA) test with a Tukey’s post-hoc test. In addition, a two-way repeated-measures ANOVA test was performed to evaluate the main effects for the condition (pre-training and post-training) and group, and the interaction effect between the condition and group. The independent variables included the groups (i.e., P, BP, and C) as the between-subjects variable and the conditions (pre-training and post-training) as the within-subjects variable. If one or more of the overall tests were significant, a follow-up test was performed to determine the simple main effects. Note that for the between-subjects variable, the one-way ANOVA with Tukey’s post-hoc test was used as the follow-up test, while for the within-subjects variable, the paired t-test was used as the follow-up test. Cohen’s *d* was used to compute the magnitude of effect size between two means. The effect size 0.1 or <0 was considered no effect; *d* = 0.2–0.4 was considered a small effect size; *d* = 0.5–0.7 was considered a moderate effect size; and *d* ≥ 0.8 was considered a large effect size [29]. The 95% confidence interval (CI) around effect size was also calculated to assess the precision of the effect size [30]. All of the statistical analyses were performed using SPSS for Windows Version 15.0 (SPSS Inc., Chicago, IL, USA). The significant level was set at *p* < 0.05.

## 3. Results

### 3.1. Demographics

No significant group differences were found in age (plyometric group: 23.20 ± 2.82 years, integrated group: 23.80 ± 4.13 years, control group: 23.50 ± 3.00 years, *p* = 0.911), body height (plyometric group: 169.30 ± 10.17 cm, integrated group: 174.40 ± 7.56 cm, control group: 170.60 ± 7.23 cm, *p* = 0.385), and body weight (plyometric group: 69.40 ± 12.41 kg, integrated group: 69.60 ± 8.64 kg, control group: 70.30 ± 9.17 kg, *p* = 0.979).

### 3.2. Joint Position Sense

**Figure 4:**
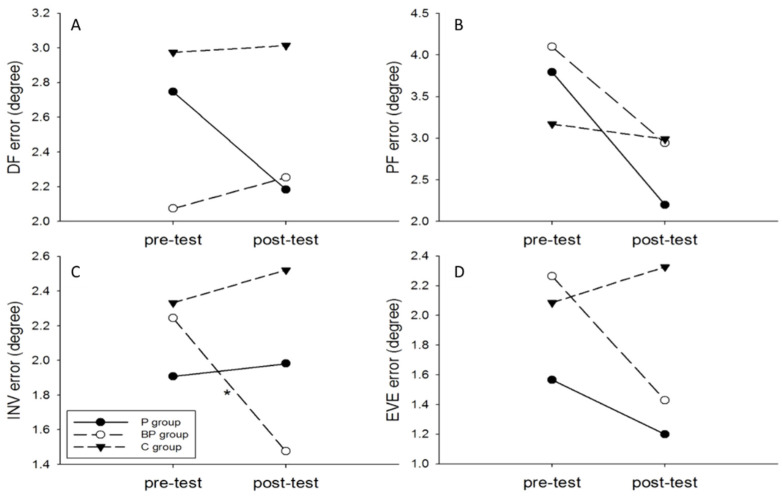
Absolute error values in ankle joint position: (**A**) 10° dorsiflexion (DF), (**B**) 15° plantar flexion, (**C**) 15° inversion (INV), and (**D**) 10° eversion. * *p* < 0.05, comparison between pre-test and post-test.

No significant interaction effect between group and condition was noted for dorsiflexion, plantarflexion, and eversion positioning error, but significant main effect for condition in plantarflexion positioning error (plyometric group: pre-training: 3.79° ± 1.98°, post-training: 2.20° ± 1.31°, t = 2.812, *p* = 0.016, Cohen’s *d* =0.947; integrated group: pre-training: 4.10° ± 1.87°, post-training: 2.94° ± 1.01°, t = 2.240, *p* = 0.045, Cohen’s *d* = 0.772) and inversion positioning error (integrated group: pre-training: 2.24° ± 1.44°, post-training: 1.48° ± 0.93°, t = 2.639, *p* = 0.022, Cohen’s *d* = 0.627) (Figure 4). The plyometric group had significantly smaller dorsiflexion and eversion joint positioning error compared to the control group (dorsiflexion: Cohen’s *d* = 1.243, 95% CI around effect size: 0.286, 2.200; eversion: Cohen’s *d* = 1.369, 95% CI around effect size: 0.395, 2.343) while the integrated group had significantly smaller inversion and eversion joint positioning error relative to the control group (inversion: Cohen’s *d* = 1.155, 95% CI around effect size: 0.208, 2.102; eversion:, Cohen’s *d* = 0.983, 95% CI around effect size: 0.055, 1.911).

### 3.3. Single-Leg Drop Landing

The group and condition interaction effects were noted on the integrated EMG of TA (F = 4.469, *p* = 0.014), GL (F = 21.434, *p* < 0.001), GM (F = 4.543, *p* = 0.013), and SOL (F = 27.025, *p* < 0.001) in the pre-landing of medial drop landing (Table 2). The results for the ANOVA indicated a significant simple main effect for the group in PL (F = 5.061, *p* = 0.008), GL (F = 6.382, *p* = 0.003), and SOL (F = 15.417, *p* < 0.001) and a significant simple main effect for condition in GL (F = 72.329, *p* < 0.001), GM (F = 20.350, *p* < 0.001), and SOL (F = 132.102, *p* < 0.001). The integrated EMG signals of the TA, GL, GM, and SOL muscles increased in the plyometric group following training, while the integrated EMG signals of the GL, GM, and SOL muscles increased in the integrated group. According to the results of post-hoc tests, both the plyometric group and integrated group had significantly greater muscle activation compared to the control group in the GL (P-C comparison: 95% CI: 9.06, 44.68, Cohen’s *d =* 1.418; BP-C comparison: 95% CI: 10.9, 53.70, Cohen’s *d =* 1.421) and the SOL (P-C comparison: 95% CI: 12.04, 41.22, effect size: 1.715; BP-C comparison: 95% CI: 13.26, 43.10, Cohen’s *d* = 1.775) (Table 2).

In the post-landing of medial drop landing, no interaction effect was found between group and condition on the integrated EMG of TA (F = 1.418, *p* = 0.248), PL (F = 1.999, *p* = 0.142) GL (F = 0.034, *p* = 0.967), GM (F = 1.264, *p* = 0.288), and SOL (F = 0.130, *p* = 0.878). The result further indicated a significant simple main effect for condition in TA in the plyometric group (plyometric group: pre-training: 102.88 ± 20.93, post-training: 119.29 ± 38.33, *p* = 0.009) (Table 3).

No significant difference was found in the adjusting time parameter for any of the training groups or muscles, other than the plantar flexor of the plyometric group (pre-training: 3.62 ± 1.37 s, post-training: 2.60 ± 1.09 s, *p* = 0.002, Cohen’s *d* = 0.824). Further analysis for the plantar flexor muscle was made and the results showed that only the soleus muscle decreased the adjusting time (pre-test: 2.85 ± 1.15 s, post-test: 1.87 ± 0.97 s, *p* = 0.006, Cohen’s *d* = 0.921).

## 4. Discussion

The aim of this study was to examine the effect of isolated plyometric training compared to the combination of integrated balance and plyometric training in recreational athletes with functional ankle instability. The results revealed that the plyometric program reduced time to stabilization during medial single-leg drop landing. Additionally, both training programs can mitigate ankle joint position sense error and improved neuromuscular control of the TA, GL, GM, and SOL during the pre-landing phase.

### 4.1. Joint Position Sense

The findings in the present study of a lower plantar flexion positioning error for both training groups and an improved inversion joint position sense for the integrated training group are consistent with the results of many previous studies [8,31]. The results suggest that plyometric exercise is beneficial in improving the proprioception of individuals with ankle instability. Note that this assertion is reasonable since plyometric training involves repetitive ballistic motions which cause the mechanoreceptors within or around the targeted joint to undergo continual stimulation [32] and therefore improve the sensitivity of the neuromuscular receptors to the joint position. The present results also imply that as compared to plyometric training, integrated training including both plyometric and balance components is a more effective strategy for improving the inversion joint position sense. Previous studies have shown that for a testing range of ± 20° from the neutral position in the sagittal and frontal planes, the absolute position sense error for the ankle joint in healthy individuals lies in the range of 1°~3° [33]. In the present study, the absolute position sense error for plantar flexion is higher than this “normative” range before training, but falls within this range following isolated plyometric training. Overall, the plyometric training and integrated balance/plyometric training improve the joint position of inversion and plantarflexion, which are critical positions to inversion ankle sprain.

With regard to the comparisons between training group and the control group in the JPS error, the plyometric group had significantly smaller errors in dorsiflexion and eversion while the integrated group had significantly smaller errors in inversion and eversion than that of the control group, with the effect sizes ranging from 0.983 to 1.369. The 95% CI around effect sizes did not include zero and this represented that the effects size estimation was precise.

### 4.2. Muscle Activation Strategy (Activation Level)

It has been reported that plyometric training affects the muscle activation level in healthy athletes [34]. Moreover, the increased muscle activation improves the functional joint stability [12]. However, the existing literature contains little information regarding the effects of plyometric training or balance training on the muscle activation in individuals with FAI. The results obtained in this study show that the activity level of the ankle plantar flexor increases during the pre-landing phase of the drop-landing task in both training groups. Moreover, the activity level of the ankle dorsiflexor (TA) increases in the post-landing phase for the plyometric group. Previous studies showed that the normalized root mean square EMG of the soleus muscle increased following four-week and eight-week plyometric training interventions in healthy participants [35]. Furthermore, for healthy individuals, exercising with exercise sandals increases the activity patterns of the lower leg muscles, particularly those of the tibialis anterior, peroneus longus, soleus, and gastrocnemius lateralis [36]. However, BOSU balance training has no significant effect on the muscle activation of the tibialis anterior, peroneus longus, and gastrocnemius medialis in healthy participants [37]. Similarly, following a six-week multi-station proprioceptive program, there is no change in the activation level of unstable ankles for the first 60 ms after a simulated sudden ankle inversion [11]. These inconsistent findings may stem from differences in the population, training type, and task evaluated [11,35]. Previous studies generally evaluated the effects of training on healthy participants with no neuromuscular impairment. Thus, it is reasonable to expect that neuromuscular adjustment/adaptation may not be strongly evident after training. In the present study, however, the medial drop-landing task was chosen for those with an unstable ankle. This task may produce challenges regarding the participants suffering from ankle sprain and present differences on training effect.

In drop-landing tasks, the pre-landing muscular activity plays an important role in determining the stability of the eccentrically-elongated tendon–muscle complex in the moments immediately after impact [38]. Additionally, the pre-activation patterns of the two-joint muscles at the knee and hip also contribute greatly toward joint stability during landing tasks [38]. In the present medial drop-landing tasks, the pre-programmed activity of the plantar flexors increased in both the plyometric training group and the integrated training group. Thus, the results suggest that during training related to balance and plyometric exercise, individuals with FAI consciously (or subconsciously) increase the pre-programmed muscle activity of the ankle joint in order to prepare for landing, cope with perturbations, and absorb the impact.

It is widely acknowledged that muscle activation prior to landing is pre-activated and is based on previous experience (i.e., feed-forward), while muscle activation subsequent to landing is reactive (i.e., feedback) [39]. However, if the preparatory muscle activity is insufficient to stabilize the joint, individuals have to rely more on the reflex mechanism of the lower extremity muscles in order to regain stability [40]. A previous study has identified a certain level of feed-forward and feedback impairment in individuals with ankle instability, and these individuals demonstrated applying a greater propulsion and braking force than healthy individuals during both planned and unplanned gait termination [41]. The increased muscle activation level in pre-landing may stem from past experience of jump exercises, while the increased muscle activity in post-landing may reflect a sensory input from the joint position sensors via feedback motor control or an increased recruitment or firing rate of the motor neurons. Plyometric training is characterized by rapid ballistic movements, and thus individuals are required to adjust their body motions in a prompt and timely manner. Individuals who perform ballistic exercise rely heavily on their previous experience (feed-forward loop) in performing the task. In other words, participants gain experience from previous training tasks, and then apply this experience in performing subsequent repetitions of the task. The present results suggest that this learning effect causes individuals with FAI to apply greater muscle activation prior to landing irrespective of the training received.

The present findings regarding the integrated EMG of the ankle evertor (peroneal longus) and invertor (tibialis anterior) in the pre-landing phase were not consistent between the two training groups. The function of the peroneus longus immediately before impact is important since the muscle should respond effectively to the inversion moment of the ankle as the foot impacts the ground [42]. A high activation level of the ankle evertor is essential in protecting the ankle at impact [43]. However, a flattening of the medial longitudinal arch occurs on contact, and thus the ankle invertor muscles (e.g., the tibialis anterior) eccentrically contract in order to control the foot and ankle complex. In this study, the muscle activity of PL did not show significant difference in the pre-landing phase. One of the possible reasons for this is that the two trainings focused on the whole lower limb performance instead of isolated muscle training. Second, the platform is 16-cm high. We selected the height in order to prevent participants from ankle injury. Therefore, it may not provide as much challenge to the PL as previous studies (30-cm high platform). Previous research has shown that the tibialis anterior plays an important role in facilitating stabilization and absorbing the impact force during closed kinetic chain activities, such as the stance phase of walking [41]. In the present study, when comparing the pre-training to the post-training in the pre-landing phase, changes in the activation level of the tibialis anterior were found in the plyometric training group; however, no change in the activation level of the tibialis anterior or peroneus longus was found in the integrated training group. Thus, it is inferred that plyometric training may be effective in stabilizing the foot and ankle via an eccentric contraction of the tibialis anterior.

In addition, both training groups demonstrated significant increase in the integrated EMG of TA, GL, GM, and SOL muscles during the pre-landing phase of medial drop-landing task when compared with the control group, with the effect sizes ranging from 0.503 to 1.775. Among them, the 95% CI around effect size in the comparisons of TA activity between the integrated group and the control group includes zero. This might be due to the effect size being less precise in smaller sample sizes or greater variabilities of the sample evaluated [30,44]. Nevertheless, our results supported that the plyometric and the integrated training both had moderate to large effects on the above-mentioned muscle activation according to the large effect size and the 95% CI provided in the majority of outcome measures.

### 4.3. Adjusting Time

The adjusting time of the ankle plantar flexor during the medial drop landing task was found to be significantly reduced in the plyometric training group. This finding is consistent with the proposed hypothesis that plyometric training improves the ability of individuals with FAI to regain stability following landing. The adjusting time parameter proposed in this study is a novel parameter for quantifying the duration of the muscle activation level following single-leg impacts and is associated with the response latency and adjustment duration following contact with the ground [45]. To date, few researchers have investigated the temporal nature of the muscle activation signal following landing from a jump. Although the time to stabilization (TTS) of the summated EMG signal in the tibialis anterior, soleus, and peroneal longus was evaluated during single-leg forward drop landings from a step, no significant difference was found between the signals of the FAI group and the control group, respectively [46]. However, in the present study, a significant reduction in the adjusting time was observed in the muscles controlling the sagittal plane ankle motion during the landing phase of the medial drop landing in the individuals who had undergone plyometric training. Thus, it appears that plyometric training can facilitate stabilization of the muscle activation by shortening the time to stabilization.

Of the various muscles evaluated in the present study, only the soleus muscle of the plyometric group showed a reduction in the adjusting time during medial drop landing. The adjusting times of the two-joint ankle plantar flexors, i.e., the medial gastrocnemius and lateral gastrocnemius, were unaffected by either plyometric training or balance training since their firing patterns are strongly associated with the knee joint motion. Future study may need to expand the sample size to increase the study power and re-evaluate the suitability of this outcome measure in this specific population.

Several limitations of the present research must be acknowledged. First, participants with bilateral ankle instability were not excluded, and testing was simply performed on the ankle joint with the lower CAIT score. Second, specific onset time of each ankle sprain was not documented and this may possibly affect their performance. Third, the number of plyometric sets and repetitions was not standardized as these were modified depending upon participants’ feedback and progress. Future studies should evaluate the effect of the plyometric training volume (number of plyometric sets and repetitions) on the ankle joint performance and prevention of ankle injury. In addition, combination between plyometric programs and other types of training such as power, strength, or endurance exercise need to be explored.

## 5. Conclusions

Isolated plyometric training and integrated balance/plyometric training both improve the ankle joint position sense of recreational athletes with FAI and increase the muscle activation level of the ankle plantar flexors during the pre-landing phase. Furthermore, isolated plyometric training leads to a more rapid stabilization in the ankle plantar flexor during a medial single-leg drop-landing task. Even though the plyometric training may be a better training type based on the present findings, both components can be selected based on the need of recreational athletes and incorporated into the training regimens and rehabilitation programs of recreational athletes with FAI. Optimal exercise prescriptions or the combination between plyometric training and other programs are highly recommended for future studies.

## Figures and Tables

**Table 1:** Subject characteristics and history of ankle sprain in each group (mean ± SD).

Subject Characteristics	Control Group	Isolated Plyometric Training Group	Integrated Balance + Plyometric Training Group	*p*-Value
CAIT score at baseline	19.90 ± 3.41	19.05 ± 2.88	17.56 ± 4.47	0.952
Total unstable ankle (bilateral:unilateral)	10 (2:8)	10 (3:7)	10 (3:7)	-
Number of ankle sprains (in 6 months)	1.70 ± 1.252	1.60 ± 0.97	1.50 ± 1.08	0.922
Number of ankle sprains (in 2 years)	4.50 ± 2.78	4.00 ± 2.54	3.50 ± 2.10	0.668

CAIT, Cumberland Ankle Instability Tool.

**Table 2:** Integrated EMG values of each group during pre-landing phase of medial drop-landing task (unit: % • ms).

Muscle	P Group		BP Group		C Group		Interaction Effect F Value	Interaction Effect *p* Value	Between- Group Effect Size, Cohen *d* (P-C Group)	Between- Group Effect Size, Cohen *d* (BP-C Group)	Between- Group Effect Size, Cohen *d* (P-BP Group)	95% CI (Effect Size P-C Group)	95% CI (Effect Size BP-C Group)	95% CI (Effect Size P-BP Group)
	Pre-Training	Post-Training	Pre-Training	Post-Training	Pre-Training	Post-Training								
TA	34.49 ± 10.98	41.89 ± 13.98 ^#^	38.46 ± 8.61	44.15 ± 16.50 *	36.88 ± 10.13	33.46 ± 12.45	4.469	0.014	0.637	0.731	−0.148	−0.261, 1.535	−0.174, 1.636	−0.730, 1.026
PL	64.24 ± 15.09	68.37 ± 26.36	70.50 ± 12.84	74.22 ± 16.05	59.62 ± 12.57	61.86 ± 14.05	0.091	0.913	0.308	0.820	−0.268	−0.574, 1.190	−0.093, 1.733	−0.612, 1.148
GL	41.33 ± 19.11	73.43 ± 21.80 *^,#^	47.21 ± 19.66	78.88 ± 28.14 *^,#^	48.39 ± 16.92	46.56 ± 15.60	21.434	<0.001	1.418	1.421	−0.217	0.202, 2.094	0.440, 2.402	−0.662, 1.096
GM	58.19 ± 24.97	74.88 ± 15.04 *^,#^	56.01 ± 30.07	69.69 ± 18.34 ^#^	61.21 ± 14.26	61.94 ± 11.77	4.543	0.013	0.958	0.503	0.310	0.033, 1.883	−0.387, 1.393	−0.572, 1.192
SOL	32.36 ± 10.06	66.66 ±16.49 *^,#^	36.23 ± 12.80	68.21 ± 17.14 *^,#^	37.81 ± 8.29	40.03 ± 14.50	27.025	<0.001	1.715	1.775	−0.092	0.690, 2.740	0.740, 2.810	−0.785, 0.970

The values presented are mean ± SD pre-landing, 200 ms prior to initial contact, *: as compared to the C group, ^#^: pre-post comparisons, P: plyometric, BP: integrated balance and plyometric, C: control, TA: tibialis anterior, PL: peroneal longus, GL: lateral gastrocnemius, GM: medial gastrocnemius, SOL: soleus.

**Table 3:** Integrated EMG values of each group during post-landing phase of medial drop-landing task (unit: % • ms).

Muscle	P Group		BP Group		C Group		Interaction Effect F Value	Interaction Effect *p* Value	Between- Group Effect Size, Cohen *d* (P-C Group)	Between- Group Effect Size, Cohen *d* (BP-C Group)	Between- Group Effect Size, Cohen *d* (P-BP Group)	95% CI (Effect Size P-C Group)	95% CI (Effect Size BP-C Group)	95% CI (Effect Size P-BP Group)
	Pre-Training	Post-Training	Pre-Training	Post-Training	Pre-Training	Post-Training								
TA	102.88 ± 20.93	119.29 ± 38.33 ^#^	94.51 ± 15.23	102.63 ± 35.58	97.32 ± 10.96	99.13 ± 23.80	1.418	0.248	0.632	0.116	0.451	−0.266, 1.530	−0.761, 0.993	−0.437, 1.339
PL	102.67 ± 25.88	114.61 ± 25.98	95.21 ± 20.21	100.96 ± 18.58	98.35 ± 18.77	94.90 ± 23.28	1.999	0.142	0.799	0.288	0.604	−0.112, 1.710	−0.593, 1.169	−0.292, 1.500
GL	87.26 ± 19.09	85.43 ± 21.58	86.04 ± 16.46	82.44 ± 21.70	92.63 ± 16.89	89.74 ± 17.40	0.034	0.967	−0.220	−0.371	0.138	−0.659, 1.099	−0.513, 1.255	−0.740, 1.016
GM	89.22 ± 14.51	92.39 ± 20.33	85.45 ± 18.75	88.26 ± 17.54	91.87 ± 12.80	86.17 ± 17.16	1.264	0.288	0.331	0.121	0.218	−0.552, 1.214	−0.756, 0.998	−0.661, 1.097
SOL	73.75 ± 24.24	77.80 ± 21.33	71.54 ± 17.61	75.68 ± 26.79	78.07 ± 17.43	79.01 ± 12.34	0.130	0.878	−0.069	−0.160	0.088	−0.808, 0.946	−0.718, 1.038	−0.789, 0.965

The values presented are mean ± SD post-landing, 200 ms prior to initial contact, ^#^: pre-post comparisons, P: plyometric, BP: integrated balance and plyometric, C: control, TA: tibialis anterior, PL: peroneal longus, GL: lateral gastrocnemius, GM: medial gastrocnemius, SOL: soleus.

## Data Availability

All data are available. Please contact the corresponding author.

## Supplementary Materials

The following are available online at https://www.mdpi.com/article/10.3390/ijerph18105269/s1, Table S1: CONSORT 2010 checklist of information to include when reporting a randomised trial; Table S2: the isolated plyometric training and the integrated balance and plyometric training programs.
